# A Preliminary Study Assessing a Transfer Learning Approach to Intestinal Image Analysis to Help Determine Treatment Response in Canine Protein-Losing Enteropathy

**DOI:** 10.3390/vetsci11030129

**Published:** 2024-03-14

**Authors:** Aarti Kathrani, Isla Trewin, Kenneth Ancheta, Androniki Psifidi, Sophie Le Calvez, Jonathan Williams

**Affiliations:** 1 Clinical Science and Services, Royal Veterinary College, Hatfield AL9 7TA, UK; 2 Pathobiology and Population Sciences, Royal Veterinary College, Hatfield AL9 7TA, UK; 3 IDEXX Laboratories Ltd., Wetherby LS22 7DN, UK

**Keywords:** machine learning, canine, gastrointestinal, diarrhea

## Abstract

**Simple Summary:**

Protein-losing enteropathy (PLE) in dogs is a condition resulting in loss of protein through the gastrointestinal tract. Protein-losing enteropathy has a guarded prognosis, with death occurring as a result of the condition in approximately 50% of dogs. This condition can be treated with diet alone or with immunosuppressant medication, and dogs treated with diet alone have a better long-term outcome. However, our ability to determine which dogs will respond to diet alone at diagnosis is limited. Therefore, the aim of our study was to determine if a machine transfer learning approach on images of intestinal biopsies collected via upper gastrointestinal tract endoscopy at diagnosis from dogs with PLE was able to predict their response to treatment. Our study showed that the model was able to differentiate intestinal biopsy images from dogs with food-responsive PLE (*n* = 7) from immunosuppressant-responsive PLE (*n* = 10) with an accuracy of 83.78%. Our results suggest that computational approaches at biopsy diagnosis may help to predict whether dogs with PLE will respond to food or immunosuppressant medication. This will help to ensure dogs with PLE are prescribed the most appropriate treatment at diagnosis to ensure optimal response and outcome.

**Abstract:**

Dogs with protein-losing enteropathy (PLE) caused by inflammatory enteritis, intestinal lymphangiectasia, or both, have a guarded prognosis, with death occurring as a result of the disease in approximately 50% of cases. Although dietary therapy alone is significantly associated with a positive outcome, there is limited ability to differentiate between food-responsive (FR) PLE and immunosuppressant-responsive (IR) PLE at diagnosis in dogs. Our objective was to determine if a transfer learning computational approach to image classification on duodenal biopsy specimens collected at diagnosis was able to differentiate FR-PLE from IR-PLE. This was a retrospective study using paraffin-embedded formalin-fixed duodenal biopsy specimens collected during upper gastrointestinal tract endoscopy as part of the diagnostic investigations from 17 client-owned dogs with PLE due to inflammatory enteritis at a referral teaching hospital that were subsequently classified based on treatment response into FR-PLE (*n* = 7) or IR-PLE (*n* = 10) after 4 months of follow-up. A machine-based algorithm was used on lower magnification and higher resolution images of endoscopic duodenal biopsy specimens. Using the pre-trained Convolutional Neural Network model with a 70/30 training/test ratio for images, the model was able to differentiate endoscopic duodenal biopsy images from dogs with FR-PLE and IR-PLE with an accuracy of 83.78%. Our study represents an important first step toward the use of machine learning in improving the decision-making process for clinicians with regard to the initial treatment of canine PLE.

## 1. Introduction

Machine learning is a subset of artificial intelligence that uses an approach to data analysis that involves building and adapting models that allow the program to learn through experience [1]. Through the use of algorithms, the machine is able to adapt its models to improve its ability to make predictions. Image recognition algorithms are specifically trained to classify images based on their content [2]. Machine learning used in image processing is usually performed using a convolutional neural network approach known as deep learning [3]. These models are trained by processing many sample images that have already been classified or annotated. Using extracted visual features of images that they have already processed in training and validation, these models improve their predictions based on rounds of training known as epochs to further refine predictions on a new test image. Machine learning can be supervised or unsupervised [1]. With supervised learning, the algorithm is trained using a dataset to recognize patterns that are associated with a specific label or annotation, for example, healthy or diseased [1]. Once the model has learned predictive patterns from the training set data, the model is then able to assign labels to the unseen test images/data [1]. In a well-trained model, the features of importance extracted/learned from each class during training will be used to generalize the test data and generate reliable predictions [1].

Machine learning has been applied with much success to cancer diagnostics in human medicine [4]. These methods are increasingly being applied to complex conditions, such as inflammatory bowel disease in humans (IBD) [5,6]. The field is expanding in human IBD, and this has been accompanied by a shift in machine learning applications from diagnostics to prognostics and the use of more complex methods, such as neural networks [6]. Studies on human IBD have used this approach on intestinal histopathology specimens for both diagnosis and prognosis [7], with one study showing its ability to predict remission or activity and outcomes in ulcerative colitis, one subtype of IBD [8].

Dogs with protein-losing enteropathy (PLE) due to inflammatory enteritis, lymphangiectasia, or both have a guarded prognosis, with death occurring as a result of the disease in approximately 50% of cases [9]. Dogs with PLE that are treated with dietary therapy alone versus diet and glucocorticoids are significantly associated with a positive outcome [10,11,12]. However, there is limited ability to differentiate between food-responsive protein-losing enteropathy (FR-PLE) and immunosuppressant-responsive PLE (IR-PLE) at diagnosis. Although one study showed a canine chronic enteropathy clinical activity index score of below 8 was able to differentiate food-responsive PLE from steroid-responsive or non-responsive PLE in dogs [12], this finding has not been replicated in other studies [10,11]. Therefore, studies investigating the utility of additional parameters to further subcategorize the different subtypes of PLE in dogs at diagnosis and, therefore, help to guide treatment decisions is needed. The objective of this study was to determine if a transfer learning computational approach to image classification on endoscopic duodenal biopsy specimens collected at diagnosis is able to differentiate FR-PLE from IR-PLE.

## 2. Materials and Methods

### 2.1. Study Design

Our study is a retrospective study. Dogs were recruited from the Queen Mother Hospital for Animals at the Royal Veterinary College. Dogs diagnosed with PLE due to chronic inflammatory enteropathy with or without lymphangiectasia on intestinal biopsy specimens collected via upper gastrointestinal tract endoscopy and that were documented to be in complete clinical and biochemical remission four months after histopathological diagnosis were eligible for inclusion. The medical records for each dog were reviewed to ensure that complete diagnostic investigations had been performed to rule out other causes of gastrointestinal signs and hypoalbuminemia. Diagnostic investigations were considered complete if they included the following: complete blood count (CBC), serum biochemistry, serum vitamin B_12_ concentration, abdominal imaging, and fecal parasitology with or without a 5-day course of fenbendazole. Serum basal cortisol concentration was measured in dogs that had changes to their CBC suggestive of hypoadrenocorticism, such as lymphocytosis or eosinophilia. An adrenocorticotropic hormone (ACTH) stimulation test was performed on those dogs with basal cortisol concentration < 2 μg/dL (<55 nmol/L) [13]. Dogs without urinalysis were included if they had pan-hypoproteinemia and low or low–normal serum cholesterol concentrations and histopathological findings compatible with a diagnosis of inflammatory enteritis with or without intestinal lymphangiectasia. All dogs included in this study had duodenal biopsy specimens collected via routine upper gastrointestinal tract endoscopy as part of their standard diagnostic investigations. Upper gastrointestinal tract endoscopy and biopsy for all dogs were performed under sevoflurane/isoflurane inhalational general anesthesia monitored by dedicated veterinary staff. Medical records and follow-ups were also reviewed to determine which treatment the dog had received prior to entering clinical and biochemical remission. Based on this information, dogs were classified as FR-PLE or IR-PLE. Cases with incomplete diagnostic investigations, those with a histopathological diagnosis of neoplasia, less than 4 months of follow-up, or that were not in complete clinical or biochemical remission at 4 months were excluded. Paraffin-embedded formalin-fixed endoscopic duodenal biopsy specimens were collected from the histopathology archive for all dogs, and repeat histopathological assessment was performed by a board-certified veterinary pathologist to verify inclusion in this study.

### 2.2. Image Pre-Processing for Machine Learning

Hematoxylin–eosin (HE)-stained 3 × 1 inch slides were scanned using a Zeiss Axio Scan.Z1 slide scanner at 20× magnification. It is currently unknown which regions within the duodenum are important in determining patient outcomes based on the treatment given. Therefore, we used the entire tissue scan for this study without specifying regions of interest. To utilize whole slide images (WSIs) for training the machine learning model, we adopted the Aachen Protocol (https://zenodo.org/record/3694994; accessed on 11 April 2023) to extract tiles from WSIs and prepared images for machine learning. Aachen protocols were applied in the development of various deep learning models, such as those used for predicting heart transplant rejection [14] and detecting Epstein–Barr virus and microsatellite instability [15], as well as predicting genetic alterations based on HE slides of gastric cancer patients [16]. We employed QuPath v0.4.3 [17] using tessellate WSIs into tiles of 512 × 512 pixels at 0.22 µm per pixel. Extracted tiles without tissue that were not centered were removed. To limit sample imbalance between classes, tile distribution was equalized based on the class with the least number of extracted tiles, in which images were randomly selected. Subsequently, the pooled images were randomly split into 50% training, 40% validation, and 10% testing.

### 2.3. Machine Learning Model Training and Hyperparameter Optimization

**Figure 1:**
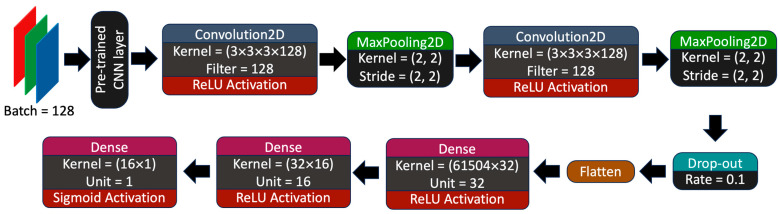
Custom convolutional neural network for model training.

The general aim of this study was to predict the binary outcome of dogs with PLE and whether the patient would benefit from diet alone or diet with immunosuppressant intervention by directly using machine learning on HE-stained duodenal images. Each model was trained on three pre-trained heads with different neural network architectures that have been previously employed to train histological images, namely MobileNetV2, InceptionV3, and EfficientNet-b7. The pre-trained head was added on top of a customized neural network layer with a total of 7 trainable layers. Input images were downsampled to 128 × 128 pixels while keeping the red-green-blue channels. The hyperparameter parameters were as follows: an Adam optimizer was used with an initial learning rate = 0.0001, which declines by a factor of 0.7 when the validation loss did not improve or change by at least three epochs (using ReducesLROnPlateau function in Keras), and binary cross-entropy for loss, and the batch size was set to 128 images per iteration and trained for 100 epochs (Figure 1).

### 2.4. Human Interpretable Machine Learning Vision

To render the machine learning vision prediction more comprehensible to human observers, we exported saliency maps onto a random tile from the testing dataset. Saliency maps could be used to visually assess pixels in tiles that could carry valuable features in predicting classes.

### 2.5. Statistical Analysis

To evaluate the learning performance of the models, loss and accuracy were plotted for training and cross-validation datasets per epoch. The loss and accuracy plot of the model were used to ‘diagnose’ and adjust the depth of the CNN to better the learning and avoid over- or underfitting. The testing dataset is the subset of data that was used to test the performance of the model; however, it was never used during training and cross-validation and represents ‘never seen’ data in the model. During generalization (i.e., FR-PLE versus IR-PLE), true positive (*TP*), true negative (*TN*), false positive (*FP*), and false negative (*FN*) were reported. To assess the classification performances of the models to predict FR-PLE versus IR-PLE on duodenal histopathology specimens at diagnosis, the following statistical indicators were used: area under the curve of the receiver operating characteristics (AUROCs), precision (Equation (1)), recall or sensitivity (Equation (2)), accuracy (Equation (3)), and *F*1 *score* (Equation (4)). Precision represents the likelihood of being truly positive among all prediction-positive samples. Recall signifies the chance of being predicted as positive within real positive samples. Accuracy pertains to the percentage of all correct predictions among the entire sample. *F*1 *score* is the harmonic mean of precision and recall values. The AUROC is calculated based on sensitivity (Equation (2)) and specificity (Equation (5)) and was generated using Keras. Both the *F*1 *score* and AUROC are measured on a scale from 0 to 1, with a higher score indicating superior model performance. These statistical indicators were previously used to compare model performances. Confusion matrices were reported, which are logs of all the predictions against the ground truth labels.

Equation (1): Precision (*Prec*)

(1) $$Prec=\frac{TP}{TP+FP}$$

Equation (2): Sensitivity/*Recall*

(2) $$Recall=\frac{TP}{TP+FN}$$

Equation (3): Accuracy (*Acc*)

(3) $$Acc=\frac{TP+TN}{TP+FP+TN+FN}$$

Equation (4): *F*1 *score*

(4) $$F1\;score=2\left(\frac{Prec\times Rec}{Prec+Rec}\right)$$

Equation (5): *Specificity*

(5) $$Specificity=\frac{TN}{TN+FP}$$

### 2.6. Computational Hardware

Training and testing were performed on a local computer server running on an AMD Ryzen Threadripper 2950X 16-Core Processor with 64 GB RAM. The estimated computing time for all the computational experiments for this study was approximately 350 CPU hours. All machine learning algorithms were completed using TensorFlow 2.11.0 (Google, Mountain View, CA, USA).

### 2.7. Carbon Impact and Offsetting

Using GreenAlgorithm v2.2 [18], we employed a carbon estimation to determine that the primary computational tasks in this study resulted in a carbon impact of 20.08 kg CO_2_ emissions (CO_2_e), equivalent to the emissions produced by 21.91 tree months. In order to compensate for this carbon footprint, we have made a commitment to plant one tree in a local forest in the UK. Over its lifespan, this tree is projected to absorb approximately 268 kg of CO_2_e, which is 26 times the amount of CO_2_e generated during this study.

## 3. Results

### 3.1. Study Population

Seventeen dogs with PLE were included in this study: seven in the FR-PLE group and ten in the IR-PLE group. Detailed serum albumin and vitamin B12 concentrations for all dogs are provided in Table 1. The median age of the dogs in the FR-PLE group was 5 years (range 3–7 years), with three male neutered and four female neutered dogs. A variety of breeds were present in this group, including a Yorkshire terrier, Shiba Inu, Cavalier King Charles Spaniel, American bulldog, Staffordshire bull terrier, Doberman, and Leonberger. The median serum albumin concentration at diagnosis in this group was 18.8 g/L (range 14.8–24.7). The histopathology of duodenal biopsy specimens collected via upper gastrointestinal tract endoscopy showed lymphoplasmacytic and neutrophilic duodenitis in two dogs, lymphoplasmacytic duodenitis in two dogs, plasmacytic duodenitis in one dog, lymphoplasmacytic and eosinophilic duodenitis in one dog, and eosinophilic and neutrophilic duodenitis in one dog. Four dogs had evidence of mild lymphangiectasia on duodenal histopathology. The median WSAVA duodenal histology score in this group was 5, with a range of 1–8. All seven dogs in the FR-PLE group were treated with a therapeutic hydrolyzed protein diet without the addition of antimicrobials or glucocorticoids. Five of the seven dogs were treated with a therapeutic hydrolyzed soy diet with 25% fat on a metabolizable energy (ME) basis, one dog was treated with a therapeutic hydrolyzed chicken liver diet with 35% fat on an ME basis, and one dog with a therapeutic hydrolyzed soy and hydrolyzed poultry liver protein diet with 42% fat on an ME basis. All dogs achieved clinical and biochemical remission and were alive at the point of follow-up at 4 months after histopathological diagnosis.

For the IR-PLE group, the median age at diagnosis was 6 years (range of 1–9 years), with four male neutered dogs, three female neutered, two female entire, and one male entire. Breeds consisted of two Bichon Frise and one each of the following: cross-breed, English bull terrier, Rottweiler, British bulldog, Staffordshire bull terrier, English pointer, Pug, and Basset Fauve de Bretagne. The median serum albumin concentration at diagnosis was 15.8 g/L (range 12.9–25.7). The histopathology of duodenal biopsy specimens collected via upper gastrointestinal tract endoscopy showed lymphoplasmacytic duodenitis in five dogs, lymphoplasmacytic and neutrophilic duodenitis in two dogs, lymphoplasmacytic, eosinophilic and neutrophilic duodenitis in two dogs, and plasmacytic duodenitis in one dog. Four dogs had evidence of lymphangiectasia: three mild and one moderate. The median WSAVA duodenal histopathology score for this group was 6, with a range of 3–11. All ten dogs received oral prednisolone at a starting dose of 2 mg/kg/day, except for one dog that started at 1.5 mg/kg/day. Four dogs received additional cyclosporin treatment, two at diagnosis, one 114 days after diagnosis, and one 28 days after diagnosis. All dogs received a therapeutic diet; eight received a hydrolyzed protein diet (seven were soy-based and one was unspecified) and two received a limited ingredient novel protein diet (one was venison and one was unspecified). All dogs achieved clinical and biochemical remission and were alive at the point of follow-up 4 months after histopathological diagnosis.

### 3.2. Tile Distribution and Training Performance

**Figure 2:**
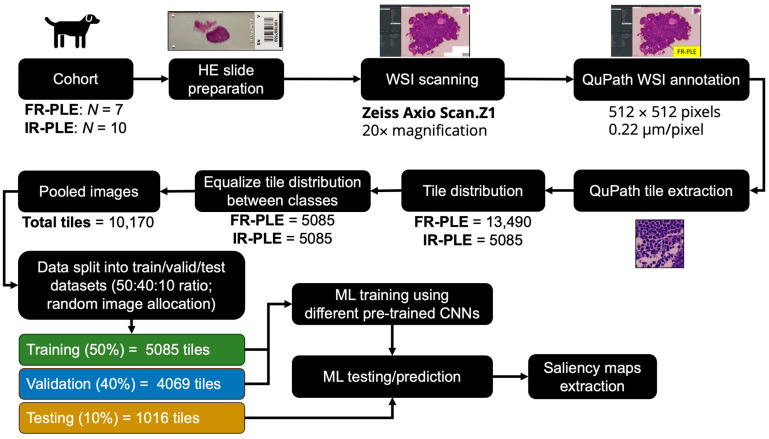
Image pre-processing and model training workflows.

A total of 13,490 and 5085 tiles were exported from FR-PLE and IR-PLE patients, respectively. Image distribution between classes was equalized to 5085 tiles per class. For the data split, we allocated 5085 (50%), 4069 and 1016 tiles to training, validation, and testing, respectively (Figure 2). To assess the performance of different pre-trained neural networks to classify FR-PLE and IR-PLE based on HE-stained duodenal sections, initial computational work was conducted using different classifiers. The selection process involved the selection of well-established architectures from TensorFlow 2.12 API (https://www.tensorflow.org/; accessed on 19 July 2023) that have been previously published in classifying HE-stained histological samples [19].

**Figure 3:**
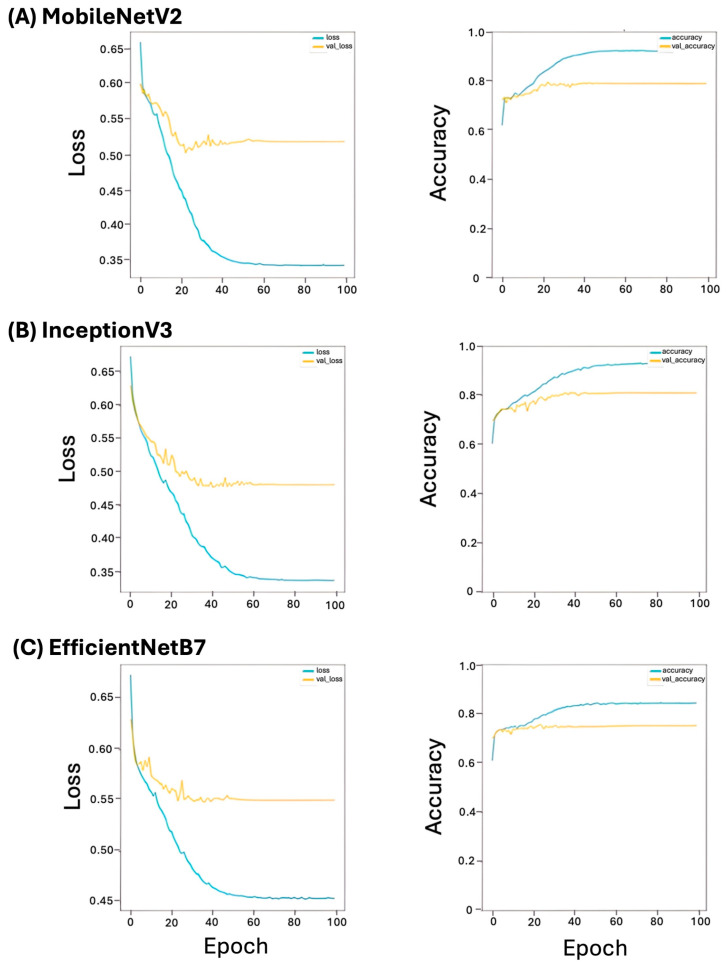
Loss and accuracy for baseline architecture during training. (**A**) MobileNetV2, (**B**) InceptionV3, and (**C**) EfficientNetB7.

Loss and accuracy were used to diagnose learning performance and to predict class against ground truth (real label), respectively. During training, the lowest calculated losses were 0.33, 0.33, and 0.45, while for validation, the losses were 0.52, 0.49, and 0.51 for MobileNetV2, InceptionV3, and EfficientNetB7, respectively (Figure 3). During training, the highest calculated accuracies were 0.91, 0.92, and 0.82, while for validation, the accuracies were 0.73, 0.79, and 0.51 for MobileNetV2, InceptionV3, and EfficientNetB7, respectively (Figure 3).

### 3.3. Model Prediction Performance

**Figure 4:**
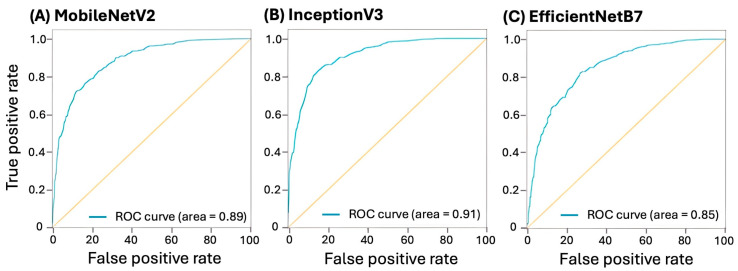
AUROC per baseline. (**A**) MobileNetV2, (**B**) InceptionV3, and (**C**) EfficientNetB7.

In terms of classification performance, there is no standardized train–validation–test split ratio for training neural networks; however, a smaller training ratio is considered stricter [19]. In Table 2, the AUROC is 0.88, 0.91, and 0.85 with F1 scores of 0.79, 0.83, and 0.76 for MobileNetV2, InceptionV3, and EfficientNetB7, respectively (Figure 4).

### 3.4. Human Explainable Machine Learning Vision

**Figure 5:**
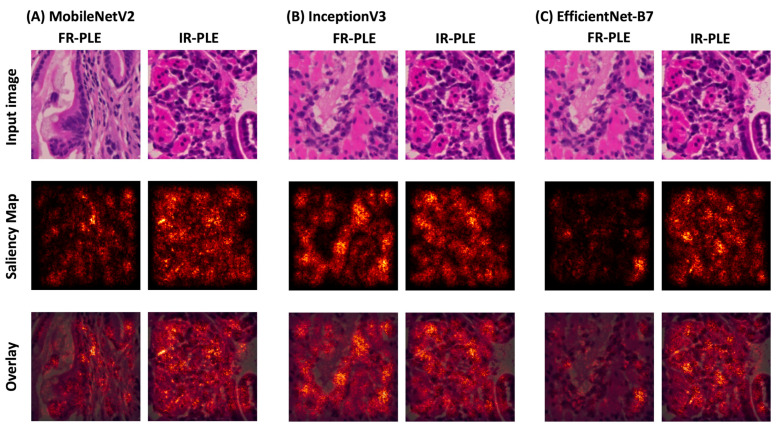
Saliency maps for FR-PLE and IR-PLE. (**A**) MobileNetV2, (**B**) InceptionV3, and (**C**) EfficientNetB7. From left to right: original image, saliency map, and the overlay of the original image and saliency map.

Extracting feature maps from the last convolutional block and the gradient scores from FR-PLE and IR-PLE classes based on the feature maps were used to generate saliency maps. Saliency maps are images in which the brightness of the pixel indicates the importance of the pixel and its significance in predicting classes in which the features/object belong or are associated. From these saliency maps, it appears that for all baseline pre-trained architecture, the amount and shape of the hematoxylin-stained regions carry significant information in classifying FR-PLE from IR-PLE or vice versa compared to eosin-stained tissue (Figure 5).

## 4. Discussion

We showed that a supervised machine transfer learning approach was able, to some extent, predict treatment response to diet alone versus diet with immunosuppressant medication by analyzing digitalized high-resolution endoscopic duodenal biopsy images collected at diagnosis with relatively good accuracy. Our study represents an important first step toward the use of machine learning in improving the decision-making process for clinicians with regard to the initial treatment of canine PLE.

Protein-losing enteropathy due to inflammatory enteritis in dogs is a chronic gastrointestinal disease with an unpredictable disease course and guarded prognosis [9,20]. Unfortunately, the exact etiology is unknown, but it is suspected to be due to a number of factors, such as genetics, mucosal immune response, intestinal microbiota, and the environment [9]. As such, the disease course for canine PLE is highly variable with varying treatment responses [20]. Dogs can experience a mild disease with a good initial treatment response or a severe refractory disease requiring many therapeutic interventions [20]. Treatment can vary from complete response to dietary therapy alone with either a hydrolyzed protein or low-fat diet to response or refractoriness to glucocorticoid treatment together with a second immunosuppressive agent [20]. Unfortunately, despite treatment, approximately 50% of dogs with PLE are euthanized due to poor response [9]. A dog’s disease course and treatment response are often unclear at diagnosis. However, several studies have shown that dogs with PLE that are responsive to dietary therapy alone have better outcomes versus dogs that are treated with concurrent glucocorticoids [10,11,12]. Therefore, the ability of a machine learning algorithm to predict dogs with PLE that will respond to dietary treatment alone at diagnosis, utilizing histopathology images from the endoscopic duodenal biopsy with good accuracy, is the first step to help strengthen the decision-making confidence of clinicians in prescribing this treatment to optimize positive outcomes for this disease. Through our promising results, machine learning could also be applied to other aspects of canine PLE, such as identifying IR-PLE from treatment non-responders, to help improve prognosis.

The histological assessment of intestinal biopsy specimens in canine chronic enteropathy is hindered by the low agreement among pathologists [21]. In order to help improve this, the WSAVA GI standardization group was formed to help standardize a list of criteria for the diagnosis and severity classification of canine chronic enteropathy [22]. Despite this, some subjectivity in histopathologic assessment still remains [23]. Therefore, the utility of machine learning to help standardize histologic assessment could help provide more accurate information for the clinician. In addition, scoring is time consuming, requires dedicated training and expertise, and more importantly, is limited by interobserver variability. Computer-aided diagnosis systems based on artificial intelligence are increasingly used to simplify and standardize the evaluation of medical imaging [4,8]. Therefore, these technologies hold promise to enhance assessment, simplify interpretation, and resolve discrepancies among pathologists. This could also help to hasten and simplify histological assessment for canine PLE.

Studies have shown that intestinal histopathology does not change after treatment and is not correlated with clinical severity in dogs with chronic enteropathy [24,25]. Similarly, the WSAVA histopathology GI scores do not correlate with outcomes in dogs with PLE [20]. Therefore, the current inflammatory and morphological criteria used for histologic diagnosis and severity of chronic enteropathy in dogs may not be optimal, as the changes do not resolve with treatment or correlate with clinical severity or outcome. Therefore, machine learning could help to prioritize which inflammatory and morphological criteria are important in the consideration of diagnosis and severity by applying algorithms to diseased versus normal tissue and tissue from different clinical severity scores. This could help to advance histopathological assessment of this disease, as well as highlight areas of importance to help uncover the pathogenesis of this disease. In line with this, a review of the saliency maps for the higher-resolution images revealed that the algorithms seemed to be putting emphasis on the crypt regions when deciding if an image was FR-PLE or IR-PLE. Further detailed review of the cryptal region in all dogs included in this study is needed to determine if this could be an area of interest in predicting treatment response in PLE. Studies have documented a discordance in WSAVA histopathology scores between the duodenum and ileum in dogs with chronic enteropathy [26,27]. As our study was preliminary, we focused on duodenal histopathology, as this is typically more accessible and universally collected during endoscopy versus ileal biopsy specimens, which can be more technically challenging to acquire via lower gastrointestinal endoscopy. However, given that ileal histopathology might be more revealing of histologic diagnosis and severity, future studies on machine learning in canine PLE should also include ileal histopathology.

The problem of uncertain prognosis and variable response to treatment is central to PLE in dogs. Therefore, machine learning could help augment the work of clinicians by providing useful information that could allow them to make better diagnostic decisions, such as the need to perform intestinal biopsies, as well as allow them to individualize medical treatment to each case. This has been exemplified in human oncology, where deep learning has been integrated into the clinical practice improving patient outcomes and accelerating clinical decision making [28]. Machine learning algorithms have also been used for predicting response to treatment and, consequently, assessing the patient’s overall prognosis and survival in human oncology [29]. Therefore, there is much potential for machine learning in the area of canine chronic enteropathy and PLE. For example, machine learning could be applied to combine gross endoscopic and histopathologic images to help determine if this combination further enhances the accuracy of predicting treatment response.

Our study had three main limitations. Firstly, the number of dogs included in our study was relatively small and, therefore, we risk our algorithm overfitting. Overfitting models have essentially ‘memorized’ the training and validation dataset, resulting in overestimated scores for accuracy; however, it risks underperformance when used to predict new data. Based on the loss vs. epoch graph, MobileNetV2, InceptionV3, and EfficientB7, we determined that the models were overfitting. In order to improve this, a veterinary community effort to acquire a sufficient number of cases to allow for more accurate machine learning models and external validation is needed. Secondly, the saliency maps for all dogs included in this study need to be reviewed in detail to identify and confirm that any areas the machine is placing emphasis on when labeling FR-PLE or IR-PLE are biologically relevant and not due to confounders, such as the intensity of staining. Thirdly, as this was a retrospective study, sequential treatment first with a therapeutic diet alone and then immunosuppressants if clinical signs persisted was not undertaken. Instead, treatment was prescribed at the discretion of the attending clinician. Therefore, it is unknown if some of the IR-PLE cases would have responded to diet alone. Prospective studies utilizing a sequential treatment approach in dogs with PLE are, therefore, needed to corroborate these findings. In addition, the retrospective design resulted in dogs with a range of serum albumin concentrations, some with relatively mild decreases being included. Therefore, this heterogeneity of clinical severity could have impacted our results.

## 5. Conclusions

In conclusion, our study showed that the supervised machine transfer learning approach could be a promising area to help predict treatment response to diet alone versus diet with immunosuppressant medication by analyzing digitalized high-resolution endoscopic duodenal biopsy images collected at diagnosis from dogs with PLE. Future directions should focus on acquiring a large dataset to mitigate the risk of the model overfitting, as well as considering an integrated approach to assess combined gross endoscopic and histologic imaging, as a way to further improve disease prediction and monitoring of this disease.

## Figures and Tables

**Table 1:** Detailed serum albumin and vitamin B12 concentrations of the dogs included in this study.

FR-PLE		IR-PLE	
Albumin (g/L)	Vitamin B12 (ng/L)	Albumin (g/L)	Vitamin B12 (ng/L)
15.8	<150	15.4	<150
23.0	<150	13.9	419
18.8	<150	13.0	184
20.7	473	19.3	<150
14.8	459	16.2	<150
24.7	233	19.6	227
18.0	WNL *	22.3	158
		25.7	298
		12.9	174
		13.7	308

* Reported as WNL (within normal limit) with exact concentration not available for review.

**Table 2:** Baseline architecture performance metrics.

Architecture	Precision	Recall	Accuracy	F1 Score	AUROC	Confusion Matrix
MobileNetV2	83.0%	79.1%	80.4%	0.79	0.88	$\left(\begin{array}{cc} 0.83 & 0.17\\ 0.23 & 0.77 \end{array}\right)$
InceptionV3	85.8%	80.9%	83.8%	0.83	0.91	$\left(\begin{array}{cc} 0.87 & 0.13\\ 0.19 & 0.81 \end{array}\right)$
EfficientNetB7	76.7%	74.6%	76.0%	0.76	0.85	$\left(\begin{array}{cc} 0.77 & 0.23\\ 0.25 & 0.75 \end{array}\right)$

AUROC—area under the curve and the receiver operating characteristics; confusion matrix—upper left = true positive, upper right = false positive, lower left = false negative, and lower right = true negative.

## Data Availability

Data are contained within the article.
